# Exploring the association of anthropometric indicators for under-five children in Ethiopia

**DOI:** 10.1186/s12889-019-7121-6

**Published:** 2019-06-14

**Authors:** Gashu Workneh Kassie, Demeke Lakew Workie

**Affiliations:** 1 Department of Statistics, Bahir Dar University, P.O. Box: 79, Bahir Dar, Ethiopia; 2 Department of Statistics, University of Gondar, P.O. Box: 96, Gondar, Ethiopia

**Keywords:** Log-linear model, Undernutrition, Stunting, Underweight, Wasting, Interaction

## Abstract

**Background:**

Child undernutrition is a global health concern. Many studies have focused on the association of childhood undernutrition indicators with their predictors. A few studies have looked at relationship between the undernutrition indicators. This study aimed at investigating the possible association structures of childhood undernutrition indicators.

**Methods:**

A log-linear model of cell counts of a three way table of stunting, wasting, and underweight was fitted based on the 2016 Ethiopia demographic health survey data. The variables of interest were generated based on the 2006 WHO Child Growth Standards as: stunted, wasted and underweight if z-scores of height-for-age, weight-for-height and weight-for age are below-2, respectively; otherwise not stunted, wasted and underweight.

**Results:**

This study showed that 36.34, 12.09 and 24.87% were stunted, wasted and underweight out of sampled children respectively and the prevalence of total undernutrition in children was about 45.96%.The fitted log-linear model showed that underweight was associated with both stunting (*P*-value< 0.001), and wasting (*P*-value< 0.001). There was no association between stunting and wasting (*P*-value = 0.999). Furthermore, the model showed that there is no a three way interaction among stunting, wasting, and underweight (*P*-value = 1.000).

**Conclusion:**

The authors conclude that there is lack of three way association of stunting, wasting, and underweight. This confirms that the three anthropometric indicators of children have multi-dimensional nature. Thus, the concerned body should consider the three undernutrition indicators simultaneously to estimate the actual burden of childhood undernourishment as they are not redundant of each other.

## Background

Child undernutrition is a global health concern. Infants and young children are the most vulnerable to undernutrition due to their high dietary requirements for growth and development. Undernutrition affects all aspects of children’s life; its effects are not limited to physical health but extend to mental, social, and spiritual wellbeing [1].

The indicators of childhood under nutrition are stunting, wasting and underweight. Stunting refers to a child who is too short for his or her age (low height-for-age); wasting refers to a child who is too thin for his or her height (low weight-for-height); and underweight refers to a child with low weight-for-age [2]. Height-for-age, weight-for-height and weight-for-age Z-scores are calculated using the 2006 WHO child growth standards recommended for international settings [3]. Children are considered stunted when they have height-for-age z-score below two compared with the WHO Child Growth standards median of same age and sex. Wasting is defined by a weight for height z-score (WHZ) below − 2 and suggests acute undernutrition or rapid weight loss. Underweight is defined by a weight for age z-score (WAZ) below − 2 [2].

The effects of childhood undernutrition are classified as short term and long-term. Mortality and morbidity are parts of short term effect as it is indicated to magnify the disease status [4]. The risk of death from childhood illness like pneumonia, diarrhea and malaria is higher for a child who is severely undernourished. The mortality of children under 5 years of age because of undernourishment is around 45%. These usually occur in developing countries [5]. The long-term effects of childhood undernourishment are linked to poor educational outcomes, unhealthy and economically unproductive adult population [6].

According to 2016 global data, 22.90 and 7.70% of children under 5 years of age are stunted and wasted, respectively [2]. Out of one third of all undernourished children under 5 years of age are found in Sub-Saharan African countries based to the 2015 MDG report. In this Sub-Saharan Africa, the prevalence of stunting, wasting and underweight for children under 5 years of age are 39%, 10 and 25%, respectively [7, 8]. These reports suggest that although undernutrition has been decreased globally, it remains a series problem in Sub-Saharan Africa.

Ethiopia has made solid progress against undernutrition in the past decade, but the country remains an extremely undernourished country. According to 2006 WHO child growth norms, 55.70% of Ethiopian preschool children were stunted in 2000. This declined to 43.4% in 2011 and to 40% in 2014 [9]. Child wasting is also relatively higher than 10%and child underweight prevalence is around 25% in 2014, down from 41% in 2000 [9]. Since children are the economic assets to the world and their future development outcomes can be influenced by their nutrition and health status, the mechanism and consequences of undernutrition and health problems need to be understood better. This is true in a country like Ethiopia where undernutrition and health problems are common.

Several studies have assessed determinants of childhood undernutrition [10–12] and have not focused on the association of stunting, wasting and underweight. A few studies tried to explore pair wise association and the three dimension association of stunting, wasting and underweight [13, 14]. Exploring the three dimension association is useful to check whether stunting, wasting and underweight have valid multidimensional nature or not. This study aimed at investigating the association of childhood undernutrition indicator variables by applying log-linear model for the three-way table to assess pair wise and three dimension association of undernutrition indicators. The interaction term would be considered to represent possible association [15].

## Methods

### Data source

This study was conducted based on the database that has been compiled as part of the 2016 Ethiopia Demographic and Health Survey (EDHS). It is the fourth Demographic and Health Survey conducted in Ethiopia [16]. The 2016 EDHS sample was stratified and selected in two stages. Each region was stratified into urban and rural areas. In the first stage, a total of 645 enumeration areas (EAs) were selected with probability proportional to EA size, of which 202 in urban areas and 443 in rural areas. An EA is a geographic area covering on average 181 households. In the second stage of selection, a fixed number of 28 households per enumeration area were selected from the newly created list of household listing using systematic sampling. In all the selected households, height and weight measurements were collected from children age 0–59 months [16]. The data set used in the analysis was Children’s Data set which was based on woman and household questionnaires. The analysis was based on children with complete anthropometric and valid age data. The overall number of child records with complete anthropometric and valid age data was 8768.

### Variables

The three anthropometric indicators are measured through z-scores for height-for-age (stunting), weight-for height (wasting) and weight-for-age (underweight) and are defined as: ${Z}_i=\frac{AI_i-\mu }{\sigma }$, where AI_i__I_ is the individual (child) anthropometric indicator, μ and σ refer respectively to median and standard deviation of the reference population [17].

The variables of interest generated were: stunted (0 = No if HAZ ≥ -2 and 1 = Yes if HAZ < - 2), wasted (0 = No if WHZ ≥ − 2 and 1 = Yes if WHZ < − 2), and underweight (0 = No if HAZ ≥ -2 and 1 = Yes if HAZ < - 2). The methodology for computing the indicators was based on the 2006 WHO Child Growth Standards [17].

### Statistics analysis

#### Log-linear model for the three-way table

A log-linear model is a statistical model for the natural logarithm of the expected frequency. The log-linear models are fitted to see the significant associations based on the cross table. Log-linear models are interpreted as generalized linear models which treat the cells counts as independent observations from the Poisson distribution with corresponding means equal to the expected cell counts. They are useful when all the three factors can be treated as response [15]. Consequently, in this study the three factors (underweight, stunting and wasting) were treated as response and the focus is on their structure of association by fitting 2^3^ + 1 = 9 log-linear possible models [14]. Therefore, the nine log-linear models of the mean cell count from the three factors are given bellow.

Note that in the last model of the following table the interactions between wasting and stunting; wasting and underweight; stunting and underweight; wasting, stunting and underweight are included.

Log-linear models are appropriate to test hypotheses about complex interactions, but the parameter estimates are less easily interpreted. Parameter estimates are log odds ratios for associations.

**Table:** 

Model	Expression	Description
Log of mean of cell counts = log(*μ*_*ijk*_)	*λ* + *λ*_*i*_^*W*^ + *λ*_*j*_^*U*^ + *λ*_*k*_^*S*^	Complete independence among the three factors (pair-wise independent). No interaction term is included.
	$\lambda +{\lambda}_i^W+{\lambda}_j^U+{\lambda}_k^S+{\lambda}_{ik}^{WS}$	Underweight is partially independent of stunting and wasting. This model contains the interaction between wasting and stunting
	$\lambda +{\lambda}_i^W+{\lambda}_j^U+{\lambda}_k^S+{\lambda}_{jk}^{US}$	Wasting is partially independent of stunting and underweight. This model contains the interaction between underweight and stunting.
	$\lambda +{\lambda}_i^W+{\lambda}_j^U+{\lambda}_k^S+{\lambda}_{ij}^{UW}$	Stunting is partially independent of wasting and underweight. This model contains the interaction between underweight and wasting.
	$\lambda +{\lambda}_i^W+{\lambda}_j^U+{\lambda}_k^S+{\lambda}_{jk}^{US}+{\lambda}_{ik}^{WS}$	Wasting & underweight are conditionally independent of stunting. This model contains the interaction terms between underweight and stunting; stunting and wasting.
	$\lambda +{\lambda}_i^W+{\lambda}_j^U+{\lambda}_k^S+{\lambda}_{ij}^{WU}+{\lambda}_{ik}^{WS}$	Stunting and underweight are conditionally independent of wasting. This model contains the interaction terms between wasting and underweight; wasting and stunting.
	$\lambda +{\lambda}_i^W+{\lambda}_j^U+{\lambda}_k^S+{\lambda}_{ij}^{WU}+{\lambda}_{jk}^{US}$	Stunting & wasting are conditionally independent of underweight. This model contains the interaction terms between wasting and underweight; underweight and stunting.
	$\lambda +{\lambda}_i^W+{\lambda}_j^U+{\lambda}_k^S+{\lambda}_{ij}^{WU}+{\lambda}_{ik}^{WS}+{\lambda}_{jk}^{US}$	Homogenous associations (every factor interacts with each other factor, but there is no interaction between all three factors)
	$\lambda +{\lambda}_i^W+{\lambda}_j^U+{\lambda}_k^S+{\lambda}_{ij}^{WU}+{\lambda}_{ik}^{WS}+{\lambda}_{jk}^{US}+{\lambda}_{ij k}^{WU S}$	All possible relationships between the factors are included.

*Key: W, U and S represent wasting, underweight and stunting respectively; λ*_*i*_^*W*^, *λ*_*j*_^*U*^, *and λ*_*k*_^*S*^ *are wasting, underweight and stunting effects respectively.*

For tables with at least three variables, unsaturated models can include association terms. Then, log-linear models are more commonly used to describe associations through two-factor terms than to describe odds through single-factor terms [18]. Interaction terms correspond to associations among variables.

#### Goodness-of-fit tests

The model with terms corresponding to all possible main effects and interactions is called the saturated model. Goodness of fit tests whether the reduced model is significantly worse than the saturated (full) model. The tests using Chi-squared statistic (*χ*^2^) and likelihood-ratio chi-squared statistic (G^2^) are goodness-of-fit tests of a log-linear model. This Goodness of Fit Tests can be used to select best fitting model. The larger the values of *G*^2^ and *χ*^2^, the more evidence exists against independence. It is also suggested by other criteria, such as minimizing AIC = − 2(Maximized log likelihood – number of parameters in the model) [18]. Data was analyzed using R version 3.5.2 and decision was done based on 0.05 levels of significance.

## Results

Table 1 shows cross tabulation of stunting, wasting and underweight. There were 36.34% stunted, 12.09% wasted and 24.87% were underweight out of sampled children.

**Table 1:** Cross classification of stunting, wasting and underweight

Wasted			Underweight		Total
			No	Yes	
No	Stunted	No	4738	125	4863
		Yes	1479	1366	2845
Yes	Stunted	No	370	349	719
		Yes	0	341	341
	Total		6587	2181	8768

**Fig. 1:**
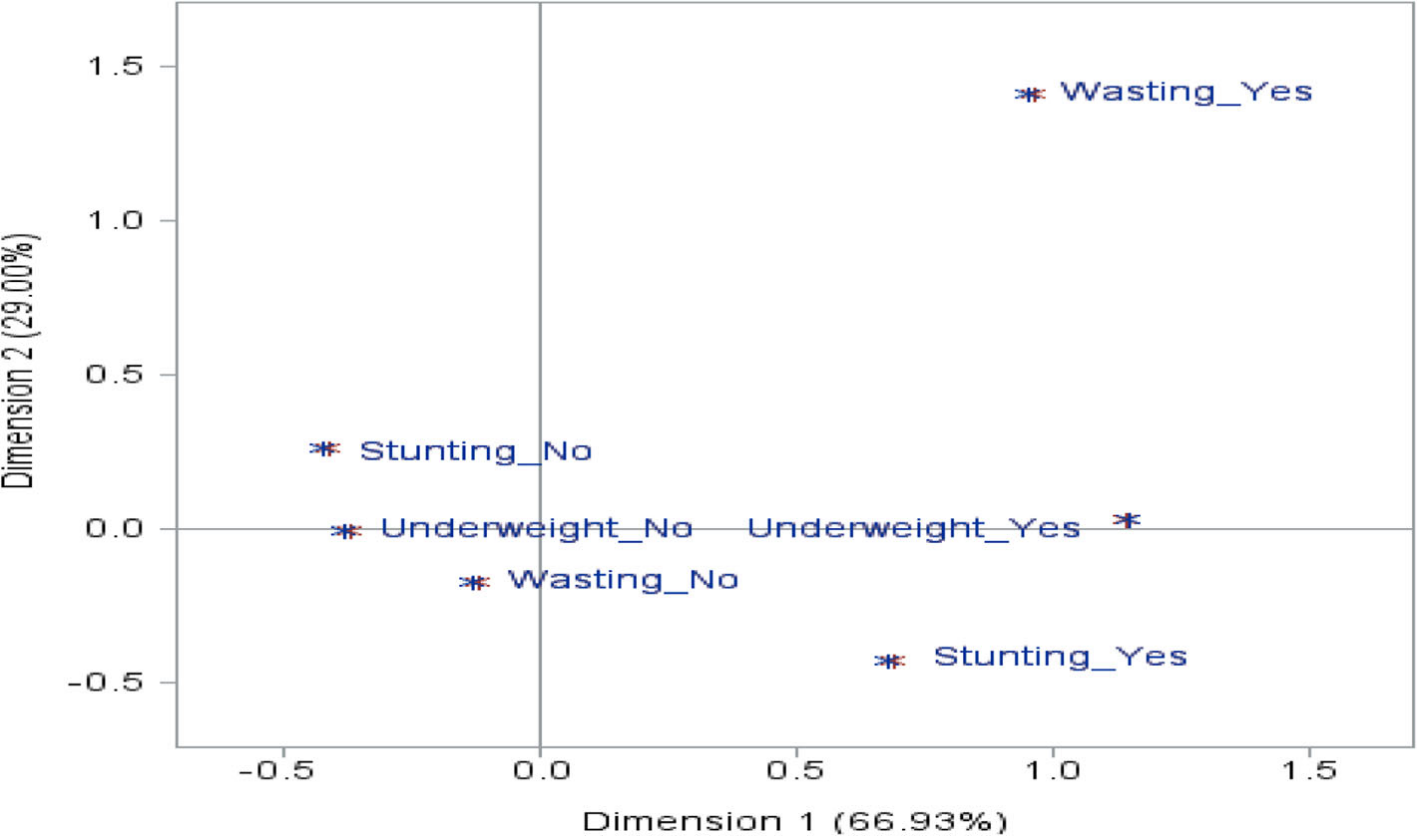
Multiple correspondence analyses of three indicators of child undernutrition

Figure 1 revealed that the multiple correspondence analysis of the three indicators of child undernutrition. There is no association between wasting and stunting as they are in the first and fourth quadrants. Whereas, underweight is located on the boundary line and indicated that it is associated with both stunting and wasting.

For measuring total prevalence of undernutrition in children, composite index of anthropometric Failure (CIAF) was followed [19]. According to CIAF classification children can be divided into: No failure; Stunted only; Underweight only; Wasted only; Stunted and underweight; Wasted and underweight; and Wasted, stunted, and underweight. By excluding children with no anthropometric failure, CIAF is a better index for estimating prevalence of total childhood undernutrition in a population [19]. Therefore, the categories of CIAF are given in Table 2 below.

**Table 2:** Categories of the Composite Indicator of Anthropometric Failure

CIAF category	Frequency (%)
No failure	4738 (54.04)
Stunted only	1479(16.86)
Underweight only	125 (1.43)
Wasted only	370 (4.22)
Stunted and underweight	1366 (15.58)
Wasted and underweight	349 (3.98)
Wasted, stunted, and underweight	341 (3.89)

Table 2 shows Categories of the Composite Indicator of Anthropometric Failure along with its frequency computed from Table 1 raw data.

According to composite index of Failure about 45.96% {=(16.86 + 1.43 + 4.22 + 15.58 + 3.98 + 3.89) %} of children were diagnosed with undernutrition while 54.04 were not diagnosed with undernutrition out of the sampled children. That is 45.96% children were stunted, underweight or wasted. In other word the prevalence of total undernutrition in children is about 45.96%. The CIAF indicates total undernutrition and does not provide any information on the prevalence of stunting, underweight and wasting relative to total undernutrition.

The likelihood ratios chi-square (G^2^), Pearson chi-square (*χ*^2^) and AIC are reported in Table 3. These are model fit test for the fitted log-linear models of cross tabulation of stunting, wasting and underweight (Table 1). The observed and fitted cell counts are compared in the fit statistics. The null hypothesis is stated as the observed and the fitted cell counts are the same (the data is well fitted by the model). The alternative hypothesis is that the observed and the fitted cell counts are different. A *P*-value less than 0.05 significant level for the fit statistics means stronger evidence against the model fits the data well and a *P*-value greater than 0.05 significant level for the fit statistics shows strong evidence that the model fits the data well.

**Table 3:** Goodness-of-fits tests for log-linear models relating stunting (S), wasting (W) and underweight (U)

Model	Log-linear model	AIC	G^2^ (Likelihood ratio)	*χ*^2^(Pearson)	df	*P*-value	
						G^2^	*χ* ^2^
1	(U, S, W)	3814.70	3748.37	3261.37	4	< 0.001	< 0.001
2	(U, SW)	3807.50	3739.18	3355.12	3	< 0.001	< 0.001
3	(W, SU)	1624.60	1556.33	2105.20	3	< 0.001	< 0.001
4	(S, UW)	2922.90	2854.67	2654.91	3	< 0.001	< 0.001
5	(SW, SU)	1617.40	1547.14	2034.31	2	< 0.001	< 0.001
6	(SW, WU)	2915.80	2845.48	2645.41	2	< 0.001	< 0.001
7	(SU, WU)	732.90	662.63	607.27	2	< 0.001	< 0.001
8	(SU, SW, UW)	91.28	19.00	10.23	1	< 0.001	0.001
9	(SWU)	74.28	0.00	0.00	0	1.000	1.000

Models 1 to 8 do not fit the data well because the *P*-values for the fit statistics are much less than 0.05 level of significance, while model 9 fits the data well as the *p*-values for the fit statistics are much greater than 0.05 level of significance (*P*-value =1.000) and has the smallest AIC value. Thus, results of the saturated model (model 9) are presented and interpreted below.

Table 4 shows results of saturated model (model 9). The null hypothesis of the individual test of coefficient of the interaction term is stated as there is no interaction between indicators of undernutrition. Thus, there is a high significant interaction between underweight and stunting since the *P*-value of the estimates of interaction term Stunting*underweight is much less than 0.05 (*P*-value< 0.001). There is also a high significant interaction between underweight and wasting (*P*-value< 0.001), but no interaction between stunting and wasting as the P-value is much greater than 0.05 (*P*-value = 0.999). Furthermore, the saturated log-linear model shows lack of three factor association among stunting, wasting and underweight (*P*-value = 1.000).

**Table 4:** Estimates for the saturated log-linear model for stunting, wasting and underweight

Coefficient	Estimate	Standard error	Z-value	*P*-value
Intercept	8.46	0.20	582.56	< 0.001
Stunting	−1.16	0.40	−39.09	< 0.001
Wasting	−2.55	0.73	−47.24	< 0.001
Underweight	−3.64	1.23	−40.12	< 0.001
Stunting*wasting	−7.35	230.62	−0.00	0.999
Stunting*underweight	3.56	1.33	36.25	< 0.001
Wasting*underweight	3.58	0.43	30.47	< 0.001
Stunting*wasting*underweight	6.70	230.68	0.00	1.000

## Discussion

In this study, a log-linear model was fitted for the three-way table in exploring association among stunting, wasting and underweight. In the log-linear model, the association of the undernutrition variables is represented by the interaction terms. Log-linear models have the advantage of assessing the three-way interaction in addition to the very common analysis of pair wise association [15]. The data is well fitted by the saturated log-linear model as the *p*-values for the fit statistics are greater than 0.05 level of significance (*P*-value =1.000) as compared to the rest of the unsaturated models.

The fitted model indicates underweight is significantly associated with stunting (P-value< 0.001). It also shows underweight and wasting are significantly interrelated (P-value< 0.001). These two findings are in line with previous studies conducted by Ngwira, Gupta and Borkotoky [13, 14]. In addition, this result is agreed on the fact that underweight is a composite measure of stunting and wasting [20, 21]. The fitted model indicates stunting and wasting are not interrelated (*P*-value = 0.999). This findings is consistent with studies done by Ngwira, Gupta and Borkotoky [13, 14]. Furthermore, the fitted model reveals there is no a three way interaction among stunting, wasting and underweight (*P*-value = 1.000). This finding is also coincides with the findings of Ngwira, Gupta and Borkotoky [13, 14]. The lack of three way interaction indicates that stunting, wasting and underweight have statistically valid multidimensional nature.

## Conclusion

The study concludes underweight is significantly associated with both stunting and wasting. The observed association of underweight with both stunting and wasting does not imply one undernutrition indicator causes the other since cross sectional data was used for the analysis. The study also concludes that stunting and wasted are not associated. On top of these, the study reveals that there is no a three way association among stunting, wasting and underweight. From the lack of three way interaction one can conclude that the anthropometric indicators of children have multidimensional nature. Thus, the concerned body should consider the three undernutrition indicators simultaneously to estimate the actual burden of childhood undernourishment as they are not redundant of each other. Finally, authors recommended that further studies can explore whether a causal relationship exists between undernutrition indicator variables or not by using data from prospective or retrospective cohort studies.

## Data Availability

The data set supporting the conclusions of this article is held by the authors and the Central Statistical Agency, CSA, Ethiopia, and the de-recognized data may be made available if a unique request is crafted from CSA website (http://www.csa.gov.et).

## Abbreviations

AIC: Akaike’s information criterion
CIAF: Composite Indicator of Anthropometric Failure
CSA: Central statistical agency
EAs: Enumeration areas
EDHS: Demographic and Health Survey
HAZ: Height-for-age z-score
MDGs: Millennium development goals
UN: United Nations
WAZ: Weight for age z-score
WHO: World health organization
WHZ: Weight for height z-score
